# Investigation of urinary volatile organic compounds as novel diagnostic and surveillance biomarkers of bladder cancer

**DOI:** 10.1038/s41416-022-01785-8

**Published:** 2022-03-29

**Authors:** Lauren Lett, Michael George, Rachael Slater, Ben De Lacy Costello, Norman Ratcliffe, Marta García-Fiñana, Henry Lazarowicz, Chris Probert

**Affiliations:** 1 Department of Molecular and Clinical Cancer Medicine, Institute of Systems, Molecular and Integrative Biology, University of Liverpool, Liverpool, L69 3GE UK; 2 School of Medicine, Cedar House, University of Liverpool, Liverpool, L69 3GE UK; 3 Centre of Research in Biosciences, University of the West of England, Frenchay Campus, Bristol, BS16 1QY UK; 4 Department of Health Data Science, University of Liverpool, Liverpool, L69 3GE UK; 5 Department of Urology, Royal Liverpool University Hospital, Liverpool University Hospitals NHS Trust, Liverpool, L7 8XP UK

**Keywords:** Diagnostic markers, Bladder cancer, Predictive markers, Molecular medicine, Diagnostic markers

## Abstract

**Background:**

The diagnosis and surveillance of urothelial bladder cancer (UBC) require cystoscopy. There is a need for biomarkers to reduce the frequency of cystoscopy in surveillance; urinary volatile organic compound (VOC) analysis could fulfil this role. This cross-sectional study compared the VOC profiles of patients with and without UBC, to investigate metabolomic signatures as biomarkers.

**Methods:**

Urine samples were collected from haematuria clinic patients undergoing diagnostic cystoscopy and UBC patients undergoing surveillance. Urinary headspace sampling utilised solid-phase microextraction and VOC analysis applied gas chromatography-mass spectrometry; the output underwent metabolomic analysis.

**Results:**

The median participant age was 70 years, 66.2% were male. Of the haematuria patients, 21 had a new UBC diagnosis, 125 had no cancer. In the surveillance group, 75 had recurrent UBC, 84 were recurrence-free. A distinctive VOC profile was observed in UBC patients compared with controls. Ten VOCs had statistically significant abundances useful to classify patients (false discovery rate range 1.9 × 10^−7^–2.8 × 10^−2^). Two prediction models were evaluated using internal validation. An eight-VOC diagnostic biomarker panel achieved AUROC 0.77 (sensitivity 0.71, specificity 0.72). A six-VOC surveillance biomarker panel obtained AUROC 0.80 (sensitivity 0.71 and specificity 0.80).

**Conclusions:**

Urinary VOC analysis could aid the diagnosis and surveillance of UBC.

## Background

### Urothelial bladder cancer

Patients with suspected bladder cancer require urological evaluation including diagnostic cystoscopy [1, 2]. In Western societies, three-quarters of primary bladder tumours are non-muscle-invasive urothelial bladder cancers (UBCs) [3]; these patients are managed endoscopically. A proportion of cases will be recurrent in nature, and some may progress to muscle invasive or metastatic disease. Therefore, lifelong endoscopic surveillance is mandated in those at risk of progression. Cystoscopy is invasive, labour-intensive, expensive and risk associated. The National Institute for Health and Care Excellence outlined the need for a urinary bladder cancer biomarker for use in surveillance, to reduce the frequency of cystoscopy [4].

### Urinary volatile organic compounds

Several studies report the diagnostic potential of urinary volatile organic compounds (VOCs) in malignancies such as bladder [5–14], prostate [15, 16] and renal cancer [17, 18]. The study of VOCs in bladder cancer has more commonly utilised pattern recognition techniques to date [5–11]; a chemical analytical approach, such as solid-phase microextraction (SPME) and gas chromatography-mass spectrometry (GC-MS), is required for the identification of individual VOC trends. SPME facilitates the isolation of VOCs, whilst GC-MS enables VOC analysis [19]. SPME-GC-MS is rapid, cost-effective and delivers reliable semi-quantitative data [20].

### Aim

This cross-sectional study compares the urinary VOC profiles of patients with and without UBC, to establish metabolomic signatures for use as diagnostic and surveillance biomarkers.

## Methods

The study flow and data analysis pipeline have been summarised in Supplementary Fig. 2.

### Participant recruitment

Patients were recruited from urology clinics at the Royal Liverpool and Broadgreen University Hospitals NHS Trust between August 1, 2016 and July 31, 2018. These patients represented two clinical opportunities: (i) diagnostic patients undergoing cystoscopy to investigate visible and non-visible haematuria; (ii) surveillance patients with previously diagnosed and treated UBC attending for follow-up cystoscopy as recommended by the current national guideline. A sample size calculation [21], assuming the recruitment ratio 1:2 (UBC cases: controls) and anticipated sensitivity and specificity of 0.90, determined 330 participants (110 cases and 220 controls) were required to achieve a 95% confidence interval width of 0.05 for the area under the receiver operating characteristic (AUROC). The study endpoint was the presence or absence of UBC. Participants were not followed-up beyond their initial cystoscopy appointment. Participants were over 18 years old. Exclusion criteria included: urinary tract infection, as cystoscopy was postponed; concurrent non-UBC malignancy, as these cases might introduce confounding VOCs; HIV infection, due to local health and safety guidance. Participants were assigned a unique identifier to maintain anonymity and allow blinding during data analysis.

Ethical approval was granted by the National Research Ethics Committee North West—Haydock (REC 14/NW/1212). Participants provided informed, written consent prior to enrolment.

### Sample collection and storage

Urine samples were collected prior to cystoscopy in the urology clinic. Samples were stored in a refrigerator until the end of the clinic and then transported to the Liverpool Tissue Bank for long-term storage at −80 °C; freezer storage of urine within 12 h of sample collection is recommended to minimise VOC alteration secondary to metabolic and degradative processes [22]. Long-term storage of urine samples at −80 °C preserves VOC signatures and promotes matrix stability [23]. Urine samples were transferred to the analytical laboratory for storage at −20 °C in the days preceding analysis.

### Chemicals, materials and instrumentation

In all, 0.3 mL 3 M sulphuric acid solution (Fisher Scientific, Loughborough, UK) was added to 0.9 mL urine in 10 mL vials (Sigma Aldrich, Dorset, UK); the acid increases the number and diversity of VOCs present in the headspace [24, 25].

A PerkinElmer Clarus 500 GC-MS single quadrupole system (PerkinElmer, Beaconsfield, UK) and PAL COMBI-xt autosampler (CTC Analytics, Switzerland) were used to analyse samples. A Zebron ZB-624 GC column (0.25-mm inner diameter, 60 m length and 1.4-µm film thickness) (Phenomenex, Macclesfield, United Kingdom) was utilised. The carrier gas was 99.996% pure helium (BOC, Sheffield, UK). A 50-µm-divinylbenzene-30-µm-carboxen-polydimethylsiloxane SPME fibre (Sigma Aldrich, Dorset, UK) was used for headspace sampling; this SPME fibre maximises the number and diversity of VOCs extracted from the headspace of urinary samples, whilst minimising the prevalence of contaminant degradation products [26]. The SPME fibre underwent pre-conditioning before use; the effectiveness of this process was assessed using blank runs.

### Volatile organic compound analysis

Samples were thawed at room temperature, then heated to 60 °C for 30 min. The SPME fibre was exposed to the headspace above the urine for 20 min and subsequently introduced to the GC injection port at 220 °C for 5 min, for VOC desorption. The GC oven was set to 40 °C with a 2-min hold time; the temperature was increased by 5 °C/min to 220 °C and held for 4 min. A solvent delay of 4 min was used. The MS system employed positive electron impact ionisation mode: scanning mass fragments 10–300 *m/z*, interscan delay 0.1 s, resolution 1000 full-width at half maximum. The helium carrier gas flowed at 1 mL/min. Samples were randomly ordered for analysis. GC-MS data were converted to NetCDF format.

### Data processing

A project-specific VOC library was built using the Automated Mass Spectral Deconvolution and Identification System (AMDIS, version 2.73, 2017) and the National Institute of Standards and Technology Mass Spectral Library (NIST-MSL, version 2.0, 2011). VOCs were added to the library when the forward and reverse match values exceeded 800, or 700 with probability >80%; this was accompanied by inspection of the fragmentation pattern in AMDIS. A unique identifier (“*tentative name, probability* = *x%, RT* = *y minutes*”) was recorded for VOCs that did not meet these criteria, to enable their review in subsequent samples. VOCs were recorded using their International Union of Pure and Applied Chemistry (IUPAC) synonym.

A batch report was generated which was re-processed with the NIST-MSL and NetCDF files using R studio (version 1.2.1335, 2019) with the Metab extension (version 1.24.0, 2011). Metab is a deconvolution software that aligns output data by assessing fragments and their retention times and then allowing a small time window to be applied to remove any compounds that fall outside of this, common in GC work [27].

### Statistical analysis

VOCs with a prevalence of <50% in all groups were excluded, as they lack discriminatory power. Missing data values were replaced with one-fifth of the VOC’s minimum level of detection. VOC data were normalised by median, log-transformed and auto-scaled for all analyses. The hypothesis of normality was rejected for all VOCs (Shapiro–Wilk test), whilst equality of variance between groups was confirmed (Levene’s test). Thereafter, the non-parametric Wilcoxon rank-sum test was applied for two-group comparisons, using Metaboanalyst (version 5.0, https://www.metaboanalyst.ca). Statistical analysis was adjusted for multiple comparison using the false discovery rate (FDR) threshold of 0.05.

Biomarker analysis was performed using Metaboanalyst. A diagnostic model was constructed using the cancer versus control dataset, whilst a surveillance model utilised the recurrence versus no recurrence dataset. Demographic and clinical participant data were not included given the exploratory nature of this research. VOC selection for inclusion in the models utilised random forest ranking, Lasso analysis and *K*-means clustering functions in Metaboanalyst. The models were built using 70% of samples and 30% were held back for internal validation. For both models, a Random Forest classifier with 100 cross-validations was applied. We report the average AUROC, accuracy (probability of correct classification), sensitivity and specificity for the training and hold-out datasets.

## Results

### Participant characteristics

A total of 305 samples were included (Supplementary Fig. 1), 96 UBC cases and 209 controls: non-UBC haematuria (*n* = 125), new UBC diagnosis (*n* = 21), no recurrence of UBC in surveillance (*n* = 84) and recurrence of UBC in surveillance (*n* = 75). Table 1 shows the participant characteristics. The median age was 70 years (interquartile range [IQR] 63–78). Kruskal–Wallis testing identified variation in age between groups (*P* = 0.021), although post hoc Bonferroni-adjusted pairwise comparisons did not reveal statistically significant results. There were slightly more male (66.2%) than female participants in the study: a male predominance was observed across all four groups, with no statistically significant difference in the male to female ratio between them (*P* = 0.059). Participant tobacco-smoking habits are summarised in Table 1; statistical analysis was limited for this variable due to the high non-disclosure rate (as discussed within the study limitations).

**Table 1:** Participant characteristics.

	Cancer		Control	
	New UBC diagnosis	Recurrence of UBC	Non-UBC haematuria	No recurrence of UBC
Number, frequency (%)				
General	21	75	125	84
Male	14 (66.7%)	54 (72.0%)	72 (57.6%)	62 (73.8%)
Female	7 (33.3%)	21 (28.0%)	53 (42.4%)	22 (26.2%)
Age median (interquartile range), years				
General	67 (60–77.5)	73 (65–80)	69 (62–76)	71.5 (65.25–81)
Male	68.5 (61–79)	73 (65.75–79)	69 (62–75.75)	73 (64.75–81)
Female	65 (58–73)	71 (62.5–84.5)	67 (60.5–77)	70.5 (65.75–83.25)
Tobacco smoking, frequency (%)				
Current smoker	4 (19.0%)	6 (8.0%)	12 (9.6%)	11 (13.1%)
Ex-smoker	8 (38.1%)	35 (46.7%)	53 (42.4%)	42 (50.0%)
Never smoker	6 (28.6%)	15 (20.0%)	52 (41.6%)	29 (34.5%)
Unknown	3 (14.3%)	19 (25.3%)	8 (6.4%)	2 (2.4%)
Indication for investigation				
Haematuria clinic diagnostic cystoscopy (visible)	17	1	61	0
Haematuria clinic diagnostic cystoscopy (non-visible)	0	0	33	0
Haematuria clinic diagnostic cystoscopy (other or unknown)	4	0	12	0
Non-haematuria clinic diagnostic cystoscopy	0	1	17	0
Cancer surveillance programme	0	73	0	84
Unknown	0	0	2	0
Procedure performed, frequency (%)				
Flexible cystoscopy (+/− biopsy)	12 (57.2%)	18 (24.0%)	114 (91.2%)	76 (90.5%)
Cystoscopy (+/− general anaesthesia + /− biopsy)	0	1 (1.3%)	2 (1.6%)	0
TURBT	9 (42.9%)	54 (72.0%)	4 (3.2%)	6 (7.1%)
Other	0	2 (2.7%)	5 (4.0%)	2 (2.4%)
Tumour stage as per TNM, frequency (%)				
Ta	9 (42.9%)	46 (61.3%)	N/A	N/A
T1	7 (33.3%)	12 (16.0%)	N/A	N/A
T2 (non-specified)	1 (4.8%)	1 (1.3%)	N/A	N/A
T2a	3 (14.3%)	5 (6.7%)	N/A	N/A
Unknown	1 (4.8%)	11 (14.7%)	N/A	N/A
Tumour grade, frequency (%)				
G1	1 (4.8%)	3 (4.0%)	N/A	N/A
G2 not specified	0	1 (1.3%)	N/A	N/A
G2 low-grade	4 (19.0%)	25 (33.3%)	N/A	N/A
G2 high-grade	2 (9.5%)	9 (12.0%)	N/A	N/A
G3	12 (57.1%)	28 (37.3%)	N/A	N/A
G3 carcinoma in situ	1 (4.8%)	1 (1.3%)		
Unknown	1 (4.8%)	8 (10.7%)	N/A	N/A

### Cancer versus control

**Fig. 1:**
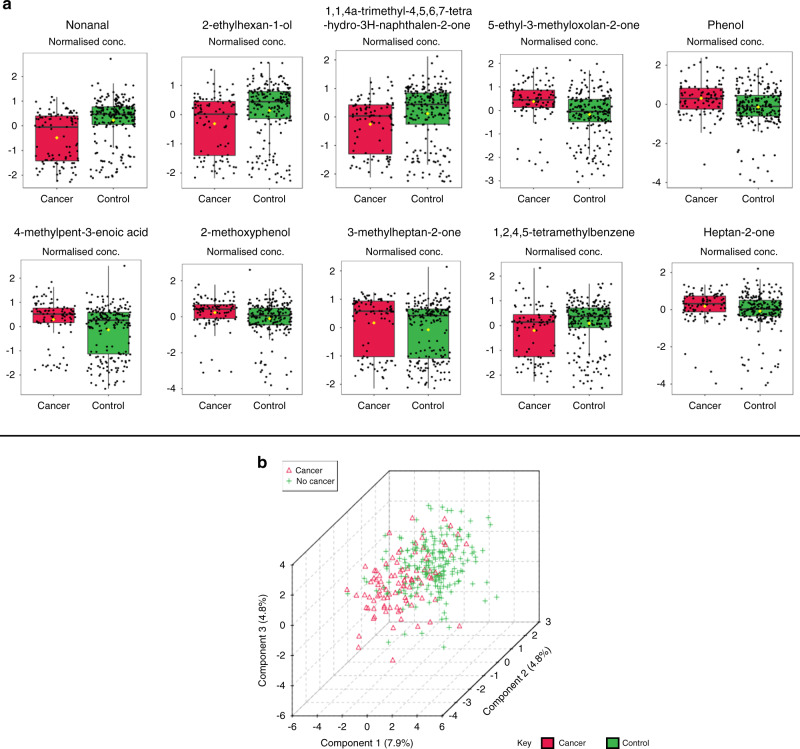
Comparison of the urinary VOC profiles of all patients with and without UBC. **a** Box and whisker plots illustrating statistically significant VOCs in UBC on Wilcoxon rank-sum testing. **b** 3D clustering of cancer versus control groups (*n* = 96 and *n* = 209, respectively) on PLS-DA.

Urinary VOC profiles of all patients with UBC (*n* = 96) and those without (*n* = 209) were compared. Wilcoxon rank-sum testing found ten VOCs that differed significantly between the two groups (Table 2 and Fig. 1a). PLS-DA showed relatively poor group separation (Fig. 1b).

**Table 2:** Statistically significant VOCs on Wilcoxon rank-sum testing of the cancer versus control comparison.

VOC	CAS number	Relative change in UBC (fold change)	Wilcoxon rank-sum *P* value	Wilcoxon rank-sum false discovery rate
Nonanal	124-19-6	Decreased (0.306)	3.78 × 10^−9^	1.85 × 10^−7^
2-ethylhexan-1-ol	104-76-7	Decreased (0.413)	2.42 × 10^−5^	3.95 × 10^−4^
1,1,4a-trimethyl-4,5,6,7-tetrahydro-3H-naphthalen-2-one	4668-61-5	Decreased (0.388)	7.81 × 10^−5^	9.57 × 10^−4^
5-ethyl-3-methyloxolan-2-one	2610-98-2	Increased (1.808)	6.36 × 10^−8^	1.56 × 10^−6^
Phenol	108-95-2	Increased (1.991)	1.94 × 10^−4^	1.66 × 10^−3^
4-methylpent-3-enoic acid	504-85-8	Increased (1.457)	2.03 × 10^−4^	1.66 × 10^−3^
2-methoxyphenol	8021-39-4	Increased (1.287)	2.60 × 10^−4^	1.82 × 10^−3^
3-methylheptan-2-one	2371-19-9	Increased (1.285)	4.68 × 10^−3^	2.67 × 10^−2^
1,2,4,5-tetramethylbenzene	95-93-2	Increased (1.119)	4.90 × 10^−3^	2.67 × 10^−2^
Heptan-2-one	110-43-0	Increased (1.177)	5.70 × 10^−3^	2.79 × 10^−2^

### No recurrence versus recurrent UBC

**Fig. 2:**
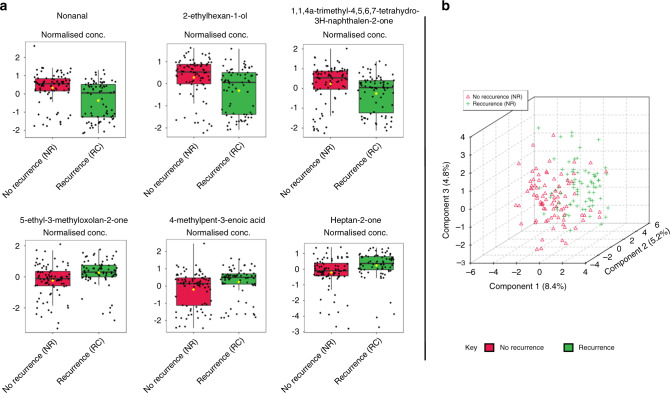
Comparison of the urinary VOC profiles of surveillance patients with recurrent and non-recurrent UBC. **a** Box and whisker plots illustrating statistically significant VOCs in the recurrence of UBC during surveillance on Wilcoxon rank-sum testing; **b** 3D clustering of no recurrence versus recurrence of UBC in surveillance (*n* = 84 and *n* = 75, respectively) on PLS-DA.

The National Institute for Health and Care Excellence recommended the development of biomarkers for surveillance. The VOC profiles of patients undergoing surveillance were compared: recurrence (*n* = 75) and no recurrence (*n* = 84) of UBC. Wilcoxon rank-sum testing demonstrated six statistically significant VOCs (Table 3 and Fig. 2a). All of these VOCs were also statistically significant in the cancer versus control comparison. Limited group separation was visualised by PLS-DA (Fig. 2b).

**Table 3:** Statistically significant VOCs in the recurrence of UBC in surveillance on Wilcoxon rank-sum testing.

VOC	CAS number	Relative change in recurrence of UBC (fold change)	Wilcoxon rank-sum *P* value	Wilcoxon rank-sum false discovery rate
**Nonanal**	124-19-6	Decreased (0.314)	*P* = 2.18 × 10^−5^	1.07 × 10^−3^
**2-ethylhexan-1-ol**	104-76-7	Decreased (0.424)	*P* = 6.50 × 10^−5^	1.24 × 10^−3^
**1,1,4a-trimethyl-4,5,6,7-tetrahydro-3H-naphthalen-2-one**	4668-61-5	Decreased (0.332)	*P* = 1.88 × 10^−4^	1.84 × 10^−3^
**5-ethyl-3-methyloxolan-2-one**	2610-98-2	Increased (1.426)	*P* = 7.57 × 10^−5^	1.24 × 10^−3^
**4-methylpent-3-enoic acid**	504-85-8	Increased (1.111)	*P* = 1.01 × 10^−3^	8.29 × 10^−3^
**Heptan-2-one**	110-43-0	Increased (1.560)	*P* = 1.44 × 10^−4^	1.77 × 10^−3^

All VOCs were significant in the cancer versus control comparison (bold).

### Non-UBC haematuria versus new UBC diagnosis

Patients presenting with haematuria will undergo a full complement of investigations, so a diagnostic biomarker for UBC is less imperative, although it may be a useful adjunct to flexible cystoscopy. Thus, VOC profiles were compared for newly presenting patients with haematuria, prior to diagnostic cystoscopy: UBC diagnosis (*n* = 21) and non-UBC haematuria (*n* = 125). The large discrepancy in group size, with the small number of new UBC diagnoses, was a potential source of inaccuracy and the comparison lacked statistical power. Wilcoxon rank-sum testing did not identify VOCs with statistically significant differences between the groups. Despite this, several of the tabulated VOC trends were similar to those observed in the cancer versus control comparison (Supplementary Table 1). In this study, visible and non-visible haematuria cases were not compared.

### Biomarker analysis

Statistically significant VOC trends in the recurrence versus no recurrence, and cancer versus no cancer, comparisons supported the hypothesis that VOCs may be of value as surveillance and diagnostic biomarkers respectively.

**Fig. 3:**
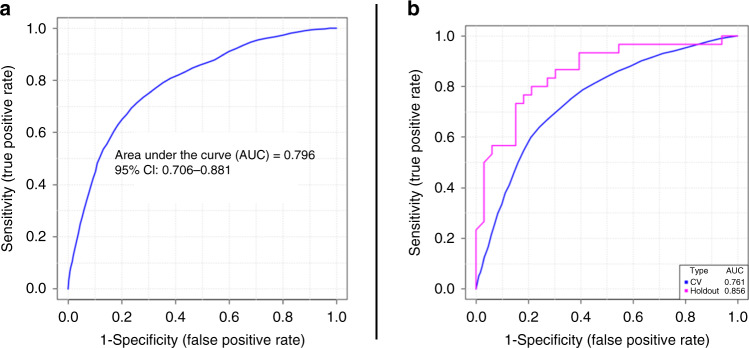
Surveillance biomarker model performance. **a** Training evaluation using the recurrence versus no recurrence dataset; **b** internal validation using the recurrence versus no recurrence comparison with a hold-out dataset for internal validation. CI confidence interval, CV cross validation.

A six-VOC surveillance biomarker model was developed using nonanal, 2-ethylhexan-1-ol, 5-ethyl-3-methyloxolan-2-one, heptan-2-one, 1,1,4a-trimethyl-4,5,6,7-tetrahydro-3H-naphthalen-2-one and propan-2-one; it had an overall AUROC of 0.80 (95% confidence interval 0.70–0.88) (Fig. 3a). Five of these VOCs were statistically significant in the recurrence versus no recurrence comparison (Table 3). The rationale for this model has been tabulated in Supplementary Table 2. The surveillance biomarker model had an average accuracy of 75% (sensitivity 0.71, specificity 0.80) when applied to the recurrence versus no recurrence dataset after 100 cross-validations. This model then underwent internal validation, achieving a hold-out AUROC of 0.86 with accuracy 78%, sensitivity 0.76 and specificity 0.80 (Fig. 3b).

An eight-VOC diagnostic model combined nonanal, phenol, 5-ethyl-3-methyloxolan-2-one, 2-ethylhexan-1-ol, 1,1,4a-trimethyl-4,5,6,7-tetrahydro-3H-naphthalen-2-one, 1-methyl-4-propan-2-ylcyclohexan-1-ol, benzaldehyde and 2,6-dimethyloct-7-en-2-ol; the overall AUROC was 0.77 (95% confidence interval 0.71–0.81)). The rationale for VOC selection in this model is described in Supplementary Table 3. Five of these VOCs were statistically significant in the cancer versus control (Table 2). The diagnostic biomarker had an average accuracy of 72% (sensitivity 0.71, specificity 0.72) when applied to the cancer versus control dataset after 100 cross-validations. This model then underwent internal validation, obtaining a hold-out AUROC of 0.80 with accuracy 72%, sensitivity 0.56 and specificity 0.87.

## Discussion

### Overview

This exploratory study investigated the urinary VOC profile of UBC using SPME-GC-MS. Statistically significant VOC trends provided some level of discrimination between patients with and without UBC. No significant differences were found between new and recurrent UBC cases (Supplementary Table 4); similar VOC patterns were observed between these groups, that differed from the controls, suggesting a distinct VOC signature for UBC. VOC-based diagnostic and surveillance biomarker models have been reported and evaluated.

### Urinary VOCs in UBC

Rodrigues et al. investigated the metabolomic signature of bladder cancer using the extracellular media of three distinct in-vitro cancer cell lines (Scaber, urinary bladder squamous cell carcinoma; J82 and 5637, UBC) [28]. The authors used principal component analysis to illustrate the separation of cancer and normal cell line groups [28]. Phenol and nonanal levels were different and the patterns were cell line-specific [28]; these VOCs also demonstrated the potential to differentiate UBC from urinary bladder squamous cell carcinoma [28]. Thus, the study of VOCs in the media of cell lines can be used to identify VOCs worthy of investigation in urine of patients. Furthermore, Rodrigues et al. found the concentration of nonanal was lower in high-grade versus low-grade disease [28], suggesting a grade-dependent relationship between the urinary volatilome and UBC; this is consistent with the significant decrease in nonanal in UBC we report in this study.

Several studies have evaluated urinary VOCs in patients with UBC [5–14]; the majority of published research reports the use of pattern recognition techniques, such as electronic nose technology, in urinary VOC analysis. A chemical analytical approach, such as GC-MS, allows a comprehensive description of the urinary volatilome. The sample sizes of previously published studies range from 10 to 277 participants [5–14]; we report data from 305 patients making this the largest study of its kind. Furthermore, this study demonstrates novelty in the metabolomic evaluation of both newly diagnosed and follow-up UBC patients. The biomarker models reported in this study achieved comparable performance to those in existing literature, further supporting the role of urinary VOC analysis.

The comparison of our findings with the existing literature is challenging due to differences in the treatment of urine samples and the analytical conditions implemented. An optimised and universal approach to urinary VOC analysis is desirable to further the evidence base and strengthen future conclusions. Tyagi et al. applied gas chromatography time-of-flight mass spectrometry to evaluate the VOC profile of untreated urinary samples [12]. The authors identified the statistical significance of nonanal in bladder cancer, although the directional trend in its abundance is not reported [12]. Pinto et al. investigated the metabolomic signature of untreated urinary samples provided by patients with bladder cancer (*n* = 53) and controls (*n* = 56) using SPME-GC-MS [13] and reported increased levels of alkanes and aromatic compounds, as well as the decreased abundance of aldehydes, ketones and monoterpenes [13]; there were no common VOCs between that research and this study. Cauchi et al. applied GC-MS to establish the UBC-VOC profile of urine samples following treatment with hydrochloric acid [14]; the significant VOCs reported in that study lacked likeness with our findings and demonstrated little similarity with the previously discussed research.

The treatment of urine samples using sulphuric acid has been shown to optimise VOC analysis by enhancing the number and diversity of potential biomarkers detectable in the headspace [24, 25]. This technique remained constant across all analyses performed within this study and facilitated the identification of distinctive VOC trends in UBC. However, the acidification process makes it difficult to establish the biological origins of urinary VOCs with confidence and some compounds might be generated in-vitro. This could be addressed by the study of VOCs in untreated urine. A comprehensive review published by Janfaza et al. provides an insight into the chemical classes and metabolic origins of cancer-associated VOCs across several biological matrices [29].

### Clinical applications of urinary VOCs in UBC

The National Institute for Health and Care Excellence recommended the development of a urinary biomarker to reduce the frequency of endoscopic UBC surveillance from annually to biennially [4]. Our surveillance biomarker model had an AUROC 0.80 (sensitivity 0.71, specificity 0.80). Therefore, 20% of negative test results would be a false negative, with a similar proportion of false positives. This model would reduce the demand for cystoscopy, at the expense of delaying the diagnosis of recurrent UBC by up to 12 months in the false negatives and unnecessarily exposing patients to the risks of cystoscopy in the false positives. There is an inherent trade-off between sensitivity and specificity: a longitudinal study of urinary VOCs and cystoscopy is required to determine the best cut-off point for clinical use. Although the sensitivity and specificity of this model are insufficient for use as a standalone investigation, these results show promise. Urinary VOC analysis may find utility in combination with more established surveillance tests.

In a research laboratory, GC-MS is labour-intensive, however, we used a Combipal system to control temperature, fibre exposure, sample extraction and injection; this reduces manual aspects of the analysis and may increase its reproducibility and precision. Alternatively, full-automated GC-sensor systems (based on the device reported by Aggio et al. [10]) should enable the workflow to be streamlined. However, such development requires significant investment and proof of concept studies, such as that reported here, are necessary to secure funding.

The diagnosis of UBC is currently reliant on the symptomatic presentation of patients. Visible haematuria is the greatest symptomatic predictor of UBC: positive predictive value 3.9% and 6.1% on first and second presentation, respectively, in patients aged over 65 years [30]. However, this is not pathognomonic of bladder cancer and there are other clinically significant causes of haematuria, so the investigation is mandated.

A meta-analysis by Chou et al. summarised the performance of established urinary biomarkers in the diagnosis and surveillance of UBC [31]. The overall sensitivities and specificities varied between assays [31]. Improved performance is observed on combining urinary biomarkers [32, 33].

### Urinary VOCs in other urological malignancies

The investigation of urinary metabolites as potential biomarkers of urinary tract disease is logical. The urinary VOC signatures of renal cancer and prostate cancer have undergone evaluation, demonstrating distinctive trends that may offer clinical utility.

Urinary VOCs have been reported in renal cell carcinoma patients [18]. This cancer shares presenting features with UBC, particularly haematuria. Wang et al. reported the significantly increased abundance of nonanal in renal cell cancer [18]; we found it in significantly decreased abundance in UBC. These contrasting results suggest nonanal may be capable of differentiating these two urological malignancies.

The urinary VOC profile of prostate cancer has also been explored. Tyagi et al. reported the statistical significance of 2-ethylhexan-1-ol and phenol amongst cases [12]. Lima et al. identified several significant prostate cancer-associated VOCs and proposed a panel of six compounds that achieved discrimination of cases from controls with an accuracy of 86% [34]. Khalid et al. reported a different set of discriminating VOCs, with a predictive model achieving an accuracy of 63–65% [9]. The prostate cancer-associated VOCs discussed in the latter two studies demonstrated no cross-over with the UBC-associated VOCs we report in this study.

### Limitations

This study aimed to identify the urinary VOC profile of UBC as diagnostic and surveillance biomarkers. The findings reported meet these predetermined aims but require external validation with larger and independent datasets.

Having benchmarked the metabolomic signature of UBC, future research should investigate its association with disease stage, grade and prognosis. Statistical analysis demonstrated no significant differences between the VOCs of patients with localised-indolent and advanced-aggressive disease in this study. These analyses were limited by the small advanced disease sample size.

We optimised our protocol to maximise the number and range of VOCs from control samples. It is possible that an alternative SPME may be better suited to specific VOCs relevant to the diagnosis of UBC.

Construction of the diagnostic model using the new UBC versus non-UBC haematuria comparison was not feasible given the small number of cancer cases; consequently, this model utilised the cancers versus control dataset in order to appraise a diagnostic biomarker capable of detecting UBC regardless of its clinical presentation.

Following exclusion of samples provided by patients with unknown smoking status, statistical analysis exhibited one significant VOC in the smoker (*n* = 33) versus non-smoker (*n* = 240) comparison: 1,1,5-trimethyl-2H-naphthalene was present in significantly decreased abundance in smokers (fold change 0.434, *P* = 8.51 × 10^−4^, false discovery rate 0.043). This compound conferred no significance in any UBC group comparisons. This comparison may have been limited by the proportionately small number of cigarette smokers; further research into the VOC profile of tobacco smoking is desirable.

Statistical analysis demonstrated that the male predominance observed within the study cohort did not differ significantly between the participant groups (*P* = 0.059). As a result, the proposed biomarker models were not adjusted for patient gender; future research may wish to evaluate the relationship between gender and VOC detection, with the view to further optimising predictive models.

Data on participant medication use and past medical history were incomplete. However, this pragmatic study recruited a large patient cohort that would accurately represent the real-world demographic variability in patients that would engage with a non-invasive biomarker test such as urinary VOC analysis. Participant exclusion based upon such factors was therefore avoided. The future interpretation of urinary VOCs in patients with co-morbidities and concurrent medication use would benefit from further research: an investigation of the relationship between medications and urinary VOCs, and a prospective study of urinary VOCs in dynamic disease states such as progressing or resolving pathophysiology.

Urine dilution provides an area of uncertainty in this research. On completion of this work, very dilute samples were excluded, and the statistical analysis repeated; their removal did not change the interpretation of the results. In future studies, it may be beneficial to place patients on a restricted diet to reduce ‘noise’ from food, and to obtain a first-pass urine collection in order to obtain more concentrated samples: however, the study was pragmatic and reflects the real world.

## Conclusion

The metabolomic signature of UBC is distinctive. Significant trends in urinary VOCs enable the identification of some but not all UBC samples from clinically relevant controls. The biomarker models proposed within this study show promise. Urinary VOC analysis may have the potential to influence future clinical practice in assisting the diagnosis and surveillance of UBC.

## Supplementary information

Supplementary material for online publication

Reproducibility Checklist

## Data Availability

The data relevant to the current study are available from the corresponding author on request.
