# Supplementary material for: Investigation of urinary volatile organic compounds as novel diagnostic and surveillance biomarkers of bladder cancer
Source: Br J Cancer. 2022 Mar 29;127(2):329–36. doi: 10.1038/s41416-022-01785-8 (PMC9296481; doi:10.1038/s41416-022-01785-8)
Supplement: Supplementary file 1 — Supplementary material for online publication [file 41416_2022_1785_MOESM1_ESM.docx]

**Supplementary Figure 1.** Sample inclusion for statistical analysis.

Participant eligible for study inclusion.

Cystoscopy procedure completed.

Urinary sample collected and analysed using SPME-GC-MS.

(n= 353)

Included for statistical analysis.

(n= 305)

Excluded (n=48)

- *Undetermined histological diagnosis (n=5)*
- *No bladder pathology (n=1)*
- *Non-UBC bladder pathology^*^ (n=6)*
- *Non-UBC malignant carcinoma^†^ (n=19)*
- *Absent NetCDF files (n=17)*

*^*^Squamous cell carcinoma (n=2), marginal lymphoma of the bladder (n=1), solitary fibrous tumour of the bladder (n=1), upper urinary tract transitional cell carcinoma (n=1), sarcomatoid tumour (n=1).*

*^†^Prostate cancer (n=18), caecal carcinoma (n=1).*

**Supplementary Figure 2:** (A) Flow diagram summarising methodological flow of urinary VOC analysis; (B) data processing and analysis pipeline.

B

Patients recruited from the Royal Liverpool and Broadgreen University Hospitals NHS Trust.

Diagnostic cystoscopy patients:

21 new UBC cases, 125 UBC-free.

Surveillance cystoscopy patients:

75 cases of recurrent UBC,

84 recurrence-free.

Written informed consent obtained.

Urine samples were collected prior to cystoscopy.

Urine samples were transported to the Liverpool Tissue Bank for storage at -80^o^C in the Liverpool Tissue Bank.

Urine samples were moved to the analytical laboratory, and stored at -20^o^C, prior to analysis in batches.

0.3mL 3M sulphuric acid solution was added to 0.9mL urine in 10mL vials.

Samples were thawed at room temperature, then heated to 60°C for 30 minutes.

The SPME fibre was exposed to the urine headspace for 20 minutes. It was then introduced to the GC injection port for VOC desorption and analysis.

A project specific VOC library was built using AMDIS and NIST-MSL.

A batch-report was generated which was re-processed using R studio with the Metab extension, producing a peak intensity table.

The resulting peak intensity table underwent statistical analysis using Metaboanalyst.

Peak intensity table

Processed VOC data

Statistical analysis

Wilcoxon rank-sum

Fold change analysis

PLS-DA

Biomarker model construction

Random forest rank

Lasso analysis

K means clustering

Biomarker model evaluation

Random forest classifier

Training set

Internal validation

Biomarker performance

AUROC

Accuracy

Sensitivity

Specificity

**Participant Recruitment**

**Urine Sample Collection**

**Urinary VOC Analysis**

**Data**

**Analysis**

A

*Abbreviations: UBC, urothelial bladder cancer; SPME, solid-phase microextraction; GC, gas chromatography; VOC, volatile organic compound; AMDIS, Automated Mass Spectral Deconvolution and Identification System; NIST-MSL, National Institute of Standards and Technology Mass Spectral Library; PLS-DA, partial least squares discriminant analysis; AUROC, area under the receiver operating characteristic.*

**Supplementary Table 1.** Comparison of the urinary VOC profiles of patients with new UBC and non-UBC haematuria.

| VOC | CAS number | Relative change in new UBC diagnosis (fold change) | Wilcoxon rank-sum p-value | Wilcoxon rank-sum false discovery rate |
| --- | --- | --- | --- | --- |
| **Nonanal** | 124-19-6 | Decreased (0.366) | p=0.002 | 0.104 |
| **2-ethylhexan-1-ol** | 104-76-7 | Decreased (0.434) | p=0.183 | 0.692 |
| **1,1,4a-trimethyl-4,5,6,7-tetrahydro-3H-naphthalen-2-one** | 4668-61-5 | Decreased (0.391) | p=0.052 | 0.523 |
| 2-methyl-5-methylsulfanylfuran | 13678-59-6 | Decreased (0.307) | p=0.763 | 0.906 |
| Propan-2-one | 67-64-1 | Increased (3.285) | p=0.656 | 0.863 |
| **5-ethyl-3-methyloxolan-2-one** | 2610-98-2 | Increased (2.546) | p=0.010 | 0.254 |
| **Phenol** | 108-95-2 | Increased (2.469) | p=0.286 | 0.692 |
| Pentan-2-one | 107-87-9 | Increased (2.193) | p=0.064 | 0.534 |
| (Methyldisulfanyl)methane | 624-92-0 | Increased (2.078) | p=0.540 | 0.799 |

Comparison of the diagnostic patient cohort identified no VOCs of statistical significance. However, nine VOCs demonstrated greater than two-fold change in the new UBC diagnosis group compared to non-UBC haematuria on fold change analysis; five of these VOCs were statistically significant in the cancer versus control comparison (as shown in the manuscript and indicated in bold in Supplementary Table 1).

**Supplementary Table 2.** The proposal and evaluation of surveillance biomarker models, based upon the recurrence versus no recurrence group comparison.

| Number of VOCs | VOCs included | AUROC  (95% confidence interval) |
| --- | --- | --- |
| 1 | Nonanal | 0.60 (0.48-0.71) |
| 2 | As above, 5-ethyl-3-methyloxolan-2-one | 0.63 (0.51 – 0.73) |
| 3 | As above, 1,1,4a-trimethyl-4,5,6,7-tetrahydro-3H-naphthalen-2-one | 0.72 (0.64 – 0.80) |
| 4 | As above, heptan-2-one | 0.74 (0.66 – 0.81) |
| 5 | As above, 2-ethylhexan-1-ol | 0.77 (0.69 – 0.85) |
| 6 | As above, propan-2-one | 0.80 (0.71 – 0.88) |
| 7 | As above, 1-methyl-4-propan-2-ylcyclohexan-1-ol | 0.79 (0.70 – 0.87) |
| 8 | As above, 1,2-xylene | 0.80 (0.71 – 0.87) |
| 9 | As above, 1-methyl-4-propan-2-yl-7-oxabicyclo[2.2.1]heptane | 0.79 (0.71 – 0.87) |
| 10 | As above, 4-methyl-1-prop-1-en-2-ylcyclohexene | 0.78 (0.70 – 0.86) |

*VOC selected for inclusion using Random Forest ranking, Lasso analysis and K-means clustering.*

**Supplementary Table 3.** The proposal and evaluation of diagnostic biomarker models, based upon the cancer versus control group comparison.

| Number of VOCs | VOCs included | AUROC  (95% confidence interval) |
| --- | --- | --- |
| 1 | Nonanal | 0.61 (0.53-0.69) |
| 2 | As above, 2-ethylhexan-1-ol | 0.64 (0.54 – 0.72) |
| 3 | As above, 5-methyl-3-ethyloxolan-2-one | 0.71 (0.63 – 0.76) |
| 4 | As above, phenol | 0.71 (0.65 – 0.77) |
| 5 | As above, 1,1,4a-trimethyl-4,5,6,7-tetrahydro-3H-naphthalen-2-one | 0.75 (0.69 – 0.80) |
| 6 | As above, 2,6-dimethyloct-7-en-2-ol | 0.76 (0.70 – 0.81) |
| 7 | As above, 1-methyl-4-propan-2-ylcyclohexan-1-ol | 0.76 (0.70 – 0.81) |
| 8 | As above, benzaldehyde | 0.77 (0.71 – 0.81) |
| 9 | As above, pentan-2-one | 0.76 (0.71 – 0.81) |
| 10 | As above, propan-2-one | 0.77 (0.72 – 0.82) |

*VOC selected for inclusion using Random Forest ranking, Lasso analysis and K-means clustering.*

**Supplementary Table 4.** Comparison of the urinary VOC profiles of newly diagnosed and recurrent UBC.

| **VOC** | **CAS number** | **Relative change in**  **new UBC**  **(fold change)** | **Wilcoxon rank-sum**  **p-value** | **Wilcoxon rank-sum false discovery rate** |
| --- | --- | --- | --- | --- |
| **1,2,4,5-tetramethylbenzene** | 95-93-2 | Decreased (0.351) | p=0.894 | 0.993 |
| **3-methylpentan-2-one** | 565-61-7 | Increased (2.803) | p=0.081 | 0.993 |
| Pentan-2-one | 107-87-9 | Increased (2.280) | p=0.202 | 0.993 |

Comparison of the two cancer groups aimed to evaluate whether the VOC profile of newly diagnosed UBC differed from recurring UBC. Three VOCs were found to differ in abundance by greater than two-fold between the groups, as presented in Supplementary Table 4. However, Wilcoxon rank-sum testing demonstrated no statistically significant difference in VOCs when patients with cancer were compared in the two groups (p-values >0.05). Two of the three VOCs identified to exhibit a notable fold-change within this analysis were statistically significant in the cancer versus control comparison (as shown in the manuscript and indicated in bold in Supplementary Table 1): 1,2,4,5-tetramethylbenzene and 3-methylpentan-2-one.
